# Supplementary material for: A Critical View of the Application of the APEX Software (Aqueous Photochemistry of Environmentally-Occurring Xenobiotics) to Predict Photoreaction Kinetics in Surface Freshwaters
Source: Molecules. 2019 Dec 18;25(1):9. doi: 10.3390/molecules25010009 (PMC7017383; doi:10.3390/molecules25010009)
Supplement: Supplementary file 1 [file molecules-25-00009-s001.zip › 3.2.4_gcc-4.4.0.zip!/3.2.4_gcc-4.4.0/3.2.4_gcc-4.4.0/doc/octave/html/interpreter/Interpolation-on-Scattered-Data.html]

Interpolation on Scattered Data - Untitled


Previous: Convex Hull,
Up: Geometry


---

### 29.4 Interpolation on Scattered Data

An important use of the Delaunay tessellation is that it can be used to
interpolate from scattered data to an arbitrary set of points. To do
this the N-simplex of the known set of points is calculated with
`delaunay`, `delaunay3` or `delaunayn`. Then the
simplicies in to which the desired points are found are
identified. Finally the vertices of the simplicies are used to
interpolate to the desired points. The functions that perform this
interpolation are `griddata`, `griddata3` and
`griddatan`.

— Function File: zi = **griddata** (x, y, z, xi, yi, method)  
— Function File: [xi, yi, zi] = **griddata** (x, y, z, xi, yi, method)  
> Generate a regular mesh from irregular data using interpolation.
> The function is defined by z `= f (`x`,` y`)`.
> The interpolation points are all `(`xi`,` yi`)`. If
> xi, yi are vectors then they are made into a 2D mesh.
>
> The interpolation method can be `"nearest"`, `"cubic"` or
> `"linear"`. If method is omitted it defaults to `"linear"`.
>
>
>
> **See also:** delaunay.

— Function File: vi = **griddata3** (x, y, z, v xi, yi, zi, method, options)  
> Generate a regular mesh from irregular data using interpolation.
> The function is defined by y `= f (`x`,`y`,`z`)`.
> The interpolation points are all xi.
>
> The interpolation method can be `"nearest"` or `"linear"`.
> If method is omitted it defaults to `"linear"`.
>
>
>
> **See also:** griddata, delaunayn.

— Function File: yi = **griddatan** (x, y, xi, method, options)  
> Generate a regular mesh from irregular data using interpolation.
> The function is defined by y `= f (`x`)`.
> The interpolation points are all xi.
>
> The interpolation method can be `"nearest"` or `"linear"`.
> If method is omitted it defaults to `"linear"`.
>
>
>
> **See also:** griddata, delaunayn.

An example of the use of the `griddata` function is

```
     rand("state",1);
     x=2*rand(1000,1)-1;
     y=2*rand(size(x))-1;
     z=sin(2*(x.^2+y.^2));
     [xx,yy]=meshgrid(linspace(-1,1,32));
     griddata(x,y,z,xx,yy);
```

that interpolates from a random scattering of points, to a uniform
grid.
